# Absence of deformed wing virus and *Varroa destructor* in Australia provides unique perspectives on honeybee viral landscapes and colony losses

**DOI:** 10.1038/s41598-017-07290-w

**Published:** 2017-07-31

**Authors:** John M. K. Roberts, Denis L. Anderson, Peter A. Durr

**Affiliations:** 1 Commonwealth Scientific and Industrial Research Organisation, Canberra, ACT 2601 Australia; 2 ADFCA, Research and Development Division, Al Ain, UAE; 3 CSIRO-Australian Animal Health Laboratory, Geelong, Victoria 3219 Australia

## Abstract

Honeybee (*Apis mellifera*) health is threatened globally by the complex interaction of multiple stressors, including the parasitic mite *Varroa destructor* and a number of pathogenic viruses. Australia provides a unique opportunity to study this pathogenic viral landscape in the absence of *V. destructor*. We analysed 1,240*A. mellifera* colonies across Australia by reverse transcription-polymerase chain reaction (RT-PCR) and next-generation sequencing (NGS). Five viruses were prevalent: black queen cell virus (BQCV), sacbrood virus (SBV), Israeli acute paralysis virus (IAPV) and the Lake Sinai viruses (LSV1 and LSV2), of which the latter three were detected for the first time in Australia. We also showed several viruses were absent in our sampling, including deformed wing virus (DWV) and slow bee paralysis virus (SBPV). Our findings highlight that viruses can be highly prevalent in *A. mellifera* populations independently of *V. destructor*. Placing these results in an international context, our results support the hypothesis that the co-pathogenic interaction of *V. destructor* and DWV is a key driver of increased colony losses, but additional stressors such as pesticides, poor nutrition, etc. may enable more severe and frequent colony losses to occur.

## Introduction

Increased awareness of the importance of pollination for functional ecosystems and global food security has seen a surge in research into pollinator health and population declines^1, 2^. Unravelling the complexity of increased honeybee (*Apis mellifera*) colony losses has been a particular focus of current research because of the economic value of honey production and crop pollination. Recent research implicates multiple stressors, including parasites, pathogens, chemicals, poor nutrition, climate and beekeeper management^3–6^. Arguably the most significant of these factors is the parasitic mite, *Varroa destructor*, which feeds on developing honeybee larvae and adult bees and transmits lethal viruses^7–11^. Since shifting host from the Asian honeybee (*A. cerana*) to *A. mellifera* in the mid-20^th^ century, this mite has spread worldwide causing significant honeybee losses^12^. The emergence of *V. destructor* has also significantly altered the viral landscape in honeybee populations globally by increasing virus transmission and causing selection of more virulent virus strains^13–16^. The combination of *V. destructor* and viruses is now considered the major cause of global colony losses^11, 13, 17, 18^, but uncovering the importance of viruses alone remains a significant challenge due to the ubiquitous presence of *V. destructor*.

Viruses typically persist as covert infections in honeybee populations, but outbreaks occur when colonies become stressed or encounter certain environmental conditions^19^. The expansion of *V. destructor* has significantly increased colony stress and elevated the importance of viruses in colony losses^11, 13, 17, 18^. This has led to increased understanding of the role of pathogenic viruses within colonies and identified deformed wing virus (DWV) as a major co-pathogen involved in colony losses in association with *V. destructor* ^17, 18, 20–22^. Although DWV was only discovered following the spread of *V. destructor*, it is thought to exist naturally at low prevalence in all honeybee populations^14, 23^. This hypothesis was recently tested following the arrival of *V. destructor* in Hawaii^14^ and New Zealand^24^, showing that DWV prevalence increased while strain diversity decreased. However, data for historical virus prevalence before *V. destructor* introduction is rare and typically predates the discovery of DWV^25, 26^.

There are now only a handful of *A. mellifera* populations in the world that are not infested by *V. destructor*. Most of these are small island populations with small-scale beekeepers. Australia is the exception by having a large commercial-scale beekeeping industry that is exposed to similar agrochemical and pathogen stressors as North America and Europe, but remains free of *V. destructor*. Australia has also not experienced the increased colony losses reported overseas. Australia’s mite-free status therefore provides an excellent stage for comparing viral landscapes with and without the confounding effects of *V. destructor*, hence providing insights into the causes of global colony losses. The last extensive studies of honeybee viruses in Australia relied on serological methods to examine apiaries during 1980 and 1983^26, 27^. This identified the presence of sacbrood virus (SBV), black queen cell virus (BQCV), Kashmir bee virus (KBV), chronic bee paralysis virus (CBPV) and cloudy wing virus (CWV). Since then honeybee virology in Australia has continued sporadically^28, 29^, but remains under-researched and molecular screening for viruses has been non-existent.

With the help of molecular methods it is possible to expand our knowledge of the diversity of viruses in Australia. Of particular note is the apparent absence of the DWV complex (DWV-A, DWV-B and DWV-C)^15, 22, 30^. There are no reported symptoms (e.g. wing deformities, shortened abdomens), but there is speculation that Australian honeybees have covert infections of DWV and recent overseas studies claim to have detected DWV-A in imported bee samples^31, 32^. There are also other viruses linked with *V. destructor* including acute bee paralysis viruses (ABPV), Israeli acute paralysis virus (IAPV) and slow bee paralysis virus (SBPV) that have not been tested for in Australia using molecular methods. Furthermore, several new viruses such as the Lake Sinai viruses (LSV1 and LSV2) have been recently identified overseas using next-generation sequencing (NGS)^33^, of which there is no information for in Australia. NGS technology has been applied in multiple insect systems for virus discovery^33–38^ and presents a valuable tool for characterising the viral landscape of Australian honeybees.

In this paper, we characterised the Australian honeybee viral landscape by both direct detection using reverse-transcription PCR (RT-PCR) and a NGS approach to deliver wider coverage and sensitivity. We hypothesised that there would be lower virus prevalence in Australian honeybees and DWV would be absent, compared to honeybee populations in the presence of *V. destructor*. Our results showed that in the absence of *V. destructor* there was still a significant prevalence and diversity of honeybee viruses, but this did not include the DWV complex or several other viruses that have been linked to *V. destructor* pathogenicity. The implications of these results for unravelling the complex epidemiology of global honeybee colony losses are discussed.

## Results

### Virus prevalence in Australian apiaries

**Figure 1:**
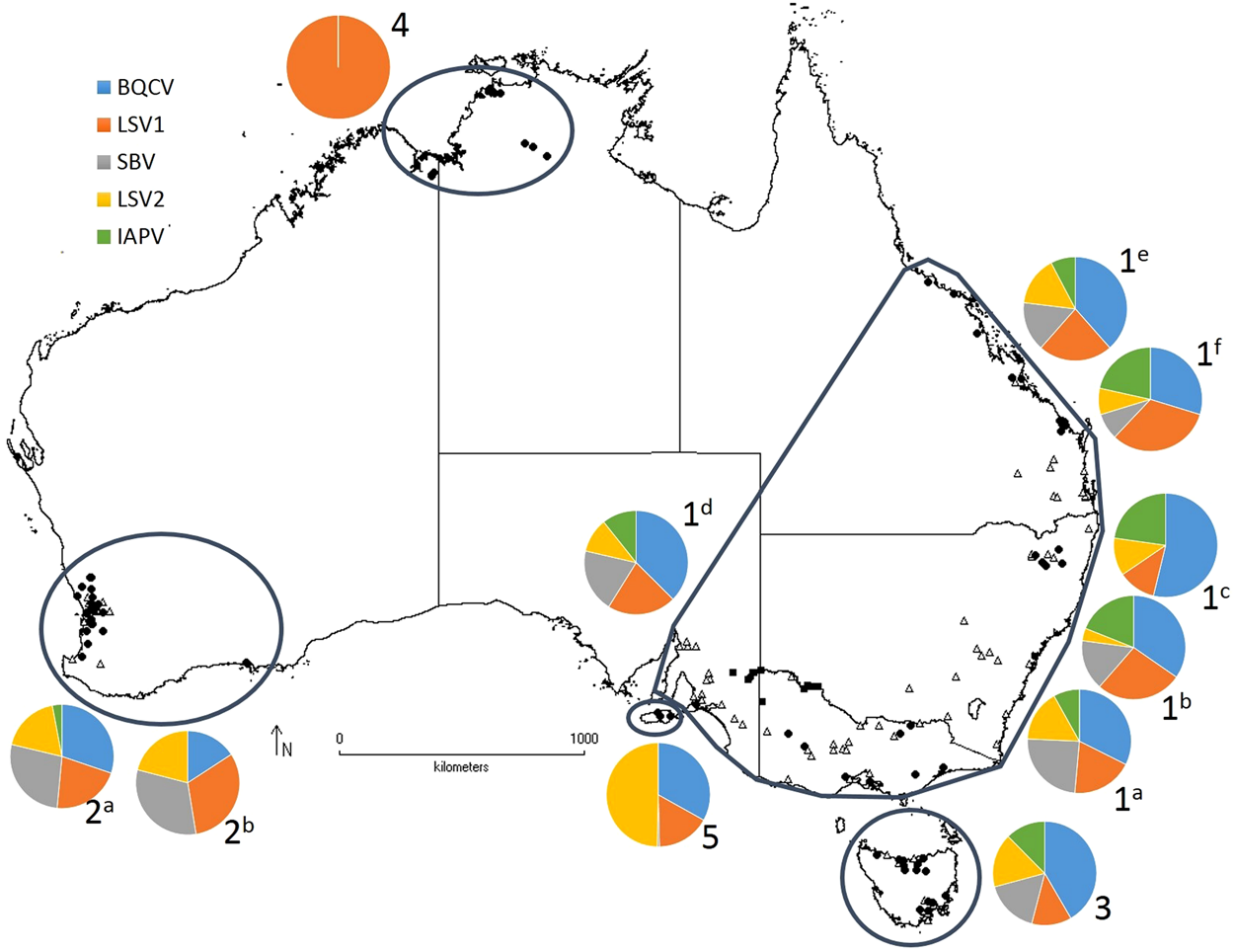
Map of Australia showing sampling sites (●) and apiary origins (▲) across five regions (1–5) and prevalence of five honeybee viruses detected by RT-PCR in adult honeybee samples. Samples for Region 1 include: a, VIC/NSW-1 August 2013; b, VIC/NSW-2 April 2014; c, VIC/NSW-3 August 2014; d, SA August 2014; e, QLD August 2013; f, QLD October 2014. Samples for Region 2 include g, WA-1 October 2013; h, WA-2 March 2014. Region 3–5 were comprised of single sampling periods. Map was created using DIVA-GIS version 7.5 (www.diva-gis.org).

Honeybees were analysed from 1,240 hives representing 155 independent apiaries across five chosen regions (Fig. 1). Five honeybee viruses were detected in adult bee samples, with BQCV being the most common virus (65%) followed by LSV1 (45%), SBV (35%), LSV2 (27%) and IAPV (21%). There was some consistency across locations and seasons, although LSV1, SBV and LSV2 were at higher prevalence in several locations (Fig. 1, Table 1). In Region 2, LSV1 and SBV had equal highest prevalence in March 2014, while LSV1 was the most common virus in Region 1 and the only virus detected in Region 4, although no viruses were found in 3 KUN samples that formed part of Region 4. In Region 5, where there are fewer managed colonies, LSV2 had highest prevalence. IAPV was notably rare outside Region 1 and 3, with only one positive sample in Region 2. An additional 27 brood samples from across all regions that were suspected of virus infection tested positive for SBV.

**Table 1:** Percentage prevalence of five honeybee viruses across Australia with the most prevalent virus in each region in bold.

Region	Location	Year	Month	*n*	BQCV	LSV1	SBV	LSV2	IAPV
1a	VIC/NSW	2013	AUG	15	**80**	47	60^1^	40	20
1b	VIC/NSW	2014	APR	17	**53**	41	24	6	29
1c	VIC/NSW	2014	AUG	18	**78**	17	0	17	33
1d	SA	2014	AUG	23	**91**	52	48^1^	26	26
1e	QLD	2014	OCT	19	58	**63**	16	16	42
1f	QLD	2013	AUG	5	**100**	60	40	40	20
2g	WA	2013	OCT	14	**71**	50	64^1^	43	7
2h	WA	2014	MAR	15	40	**80**	**80**	53	0
3	TAS	2014	NOV	15	**67**	20	27^1^	27	20
4a	NT	2014	JUN	7	0	**43**	0	0	0
4b	KUN	2014	JUN	3	0	0	0^1^	0	0
5	KI	2014	APR	4	50	25	0^1^	**75**	0
	Total			155	65	45	35	27	21

^1^Brood sample positive for SBV.

Overall, virus infections were very common in Australian honeybees with only 14% of samples free of the target viruses. Multiple viruses were detected in the majority of samples (61%), with four viruses co-infecting between 3% and 17% of samples in Regions 1 and 2 and all five viruses detected in one Region 1 sample (SA). However, five other honeybee viruses were not detected in any region; DWV, SBPV, ABPV, KBV and CBPV, which included an additional 124 brood samples tested for DWV and SBPV.

### NGS of Australian honeybee populations

NGS was used to gain a more complete picture of the honeybee virus landscape in Australia by identifying virus genomes in nine pooled adult bee samples from Regions 1–4. This approach increased our sensitivity for detecting low prevalence viruses and avoided potential problems with primer specificity. All five viruses detected by RT-PCR were identified in each pooled sample, with a high number of reads covering the full genome in most cases. Normalised read counts also provided an indication of relative infection levels for each virus (Table 2). Consistent with the prevalence data, BQCV was commonly the most abundant virus with SBV and IAPV having high read counts in some samples. LSV1 and LSV2 were generally least abundant suggesting few overt infections from these viruses. Interestingly, NGS revealed all five viruses in the NT sample when only LSV1 was detected by RT-PCR. LSV1 had the highest read count (490,875 reads) while the other viruses had approximately 30,000 reads between them.

**Table 2:** Normalised reads of honeybee viruses detected from Region 1 to 4 defined in Fig. 1 with the most prevalent virus in each region in bold.

Sample	BQCV	LSV1	SBV	LSV2	IAPV	DWV^1^	SBPV^2^	CBPV
VIC/NSW-1	**24.7M**	101,677	6.3M	175,440	550,000	116	0	0
VIC/NSW-2	7.6M	87,469	9.8M	59,982	**24.7M**	4	15	1
VIC/NSW-3	**21.2M**	215,972	155,404	1.3M	9.7M	1	8	0
SA	6.8M	3.6M	**9.3M**	1.3M	3.4M	0	0	0
QLD	**21.9M**	1M	572,995	509,751	9.2M	211	38	3
WA-1	**35.1M**	2,666	1M	24,957	2,837	0	3	0
WA-2	**22.2M**	11,794	7.3M	242,115	3,605	141	9	0
TAS	**30.4M**	499,235	4.7M	116,419	55,483	2	1	1
NT	6,199	**490,875**	4,258	17,897	2,622	991	88	0

Reads for DWV and SBPV strains are mapped with a similarity threshold of 0.7 and all other viruses are mapped with a similarity threshold of 0.9.

^1^virus complex including DWV-A, DWV-B and DWV-C master variants.

^2^virus complex including SBPV-Rothamstead and SBPV-Harpenden.

A small number of sequence reads mapped to the DWV and SBPV and CBPV reference genomes when using a lower similarity threshold of 0.7 (Table 2). Manual inspection of these reads revealed only short sequence fragments (<200 nucleotides) aligned to the reference genomes with variable sequence similarity (Supplementary material 2). For example, 15 sequence fragments aligned to all three DWV strains with an overall similarity of 69–74% (Supplementary material 3). This level of identity from only limited sequence fragments is insufficient to support the presence of these viruses, despite both DWV and SBPV existing as virus complexes with 16–20% variation^22, 39^. A number of sequence reads also mapped to the KBV reference genome, but still had higher identity with IAPV.

### Australia’s honeybees are unique on the colony stress spectrum

A comparison of 41 similar virus studies highlighted the unique stress profile of Australia’s honeybee population (Supplementary material 4). No other *A. mellifera* population has been identified that has confirmed the absence of *V. destructor* and DWV, and also has a large commercial-scale beekeeping industry. We distilled these studies into five stress profiles representing the spectrum of global *A. mellifera* populations to show that *V. destructor* and DWV are key factors in increased colony losses (Table 3).

**Table 3:** Five stress profiles identified across comparable studies of global *A. mellifera* populations compiled as part of a semi-systematic review (see Supplementary material 4).

Stress profile	Colony stressors				Increased colony losses reported	Identified populations	Commercial-scale beekeepers
	*Varroa destructor*	DWV complex	Other pathogens	Abiotic stressors			
1	No	No	Most	Most	No	Australia	Yes
2	No	No	Few	Few	No	Uganda, Norfolk Island	No
3	No	Yes	Few	Few	No	Newfoundland Island	No
4	Yes	Yes	Some/Most	Some/Most	No	Europe (some countries). New Zealand, South America, Africa, Asia	Yes (most countries)
5	Yes	Yes	Most	Most	Yes	North America, Europe (some countries)	Yes

Profiles are characterised by the presence or absence of known or hypothesized causes of increased honeybee colony losses. In relation to Fig. 4, populations with profiles 1–3 are on the lower end of the stress spectrum, populations with profile 4 range across the middle of the stress spectrum and populations with profile 5 are on the high end of the stress spectrum.

### Diversity of honeybee viruses

**Figure 2:**
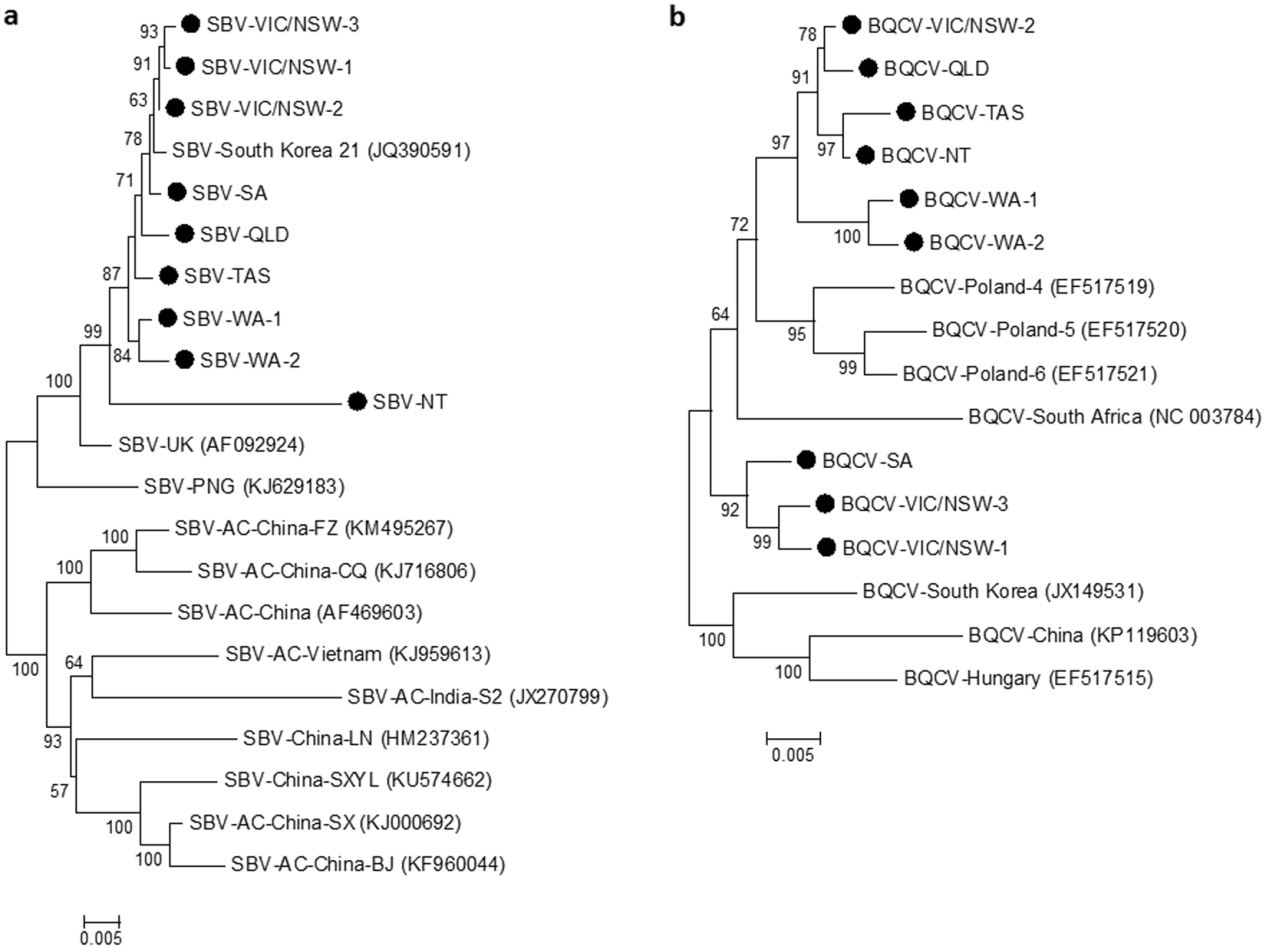
Maximum likelihood consensus tree of (**a**) SBV isolates based on 2,761 amino acids where AC indicates isolates obtained from *A. cerana* and (**b**) BQCV isolates based on 2,412 amino acids. Isolates from this study are indicated by (●).

Phylogenetic analysis of consensus genomes revealed considerable diversity between sampling regions and Australian isolates typically formed distinct clades from overseas isolates. BQCV isolates from this study were split into two distinct clades, where the winter isolates from VIC/NSW and SA grouped separately from the remaining Australian isolates (Fig. 2a). Similarly, SBV isolates formed several distinct clusters, with those from VIC/NSW and SA having greater similarity to a Korean isolate from *A. mellifera* than the original UK reference strain (Fig. 2b).

**Figure 3:**
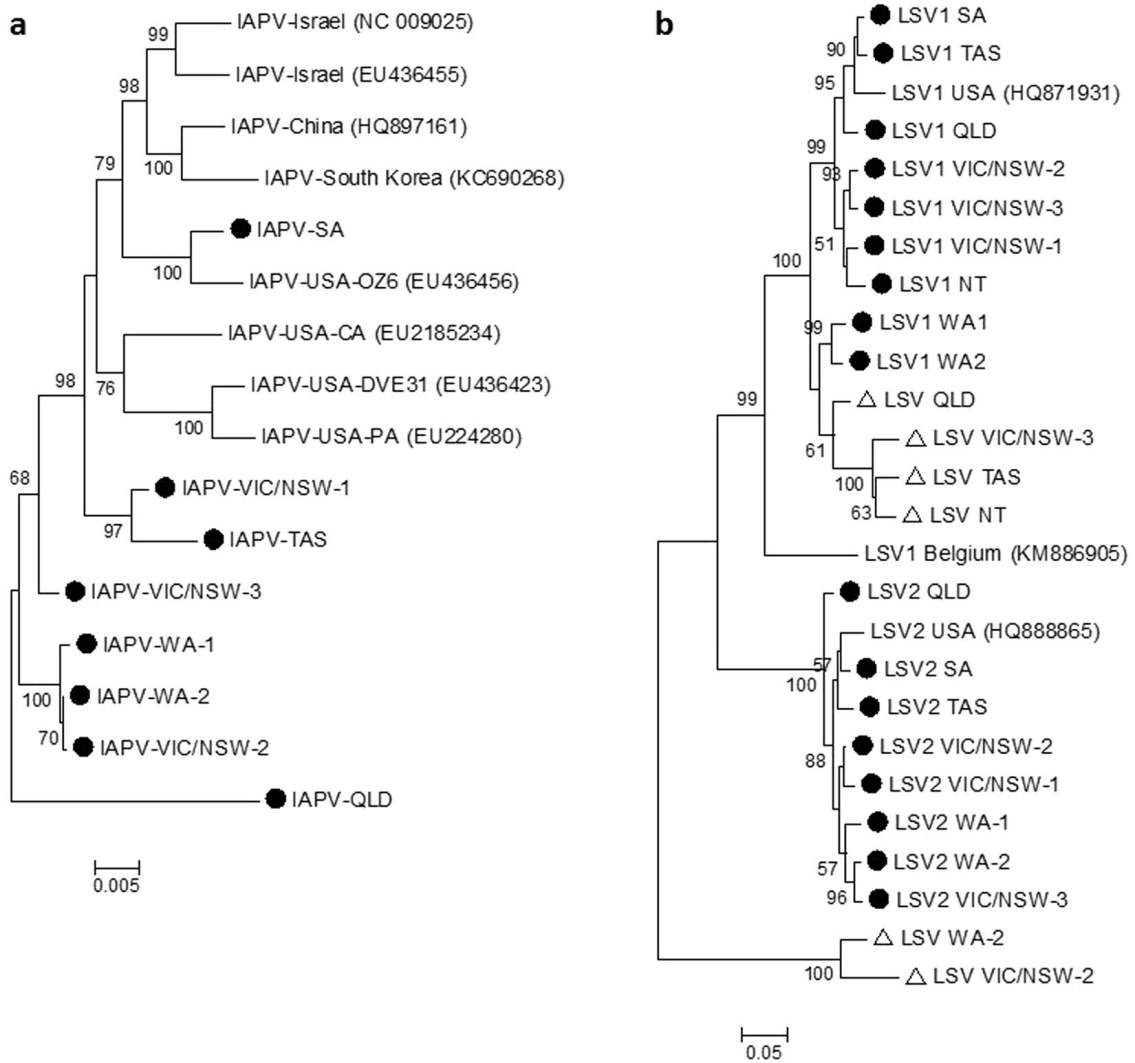
Maximum likelihood consensus tree of (**a**) IAPV isolates based on 2,849 amino acids and (**b**) LSV isolates based on 600 amino acids of the RdRP gene. Isolates of IAPV, LSV1 and LSV2 from this study are indicated by (●) and variant LSV isolates from this study are indicated by (▲). The NT isolates were excluded because of low genome coverage.

IAPV has been implicated in colony losses in Israel and the USA, and Australian honeybees have been suggested as the source population. Interestingly, only the SA isolate grouped closely with an overseas strain, which was a USA isolate obtained from imported Australian bees (Fig. 3a). Remaining Australian isolates were split into two clades. The VIC/NSW-1 and TAS isolates were positioned among USA isolates, while the other isolates branched together as an outside clade. Therefore despite potential pathways through trade of live bees, this result does not indicate that Australian honeybees are the source population for IAPV.

LSV1 and LSV2 are recently described viruses from the USA that have since been detected in Europe and have been also correlated with colony losses. The SA and TAS isolates were most similar to the original USA strains, while the remaining isolates separated into several distinct groups with some geographic clustering (Fig. 3b). *De novo* assembly also revealed the presence of multiple variant LSV strains. Partial genomes were recovered from all samples that shared approximately 70% identity to LSV1 and LSV2. Other LSV strains (LSV3–LSV7) with similar levels of divergence have also been identified from Europe and the USA^40–42^ and from two previous Australian studies^43, 44^. Only partial gene sequences are available for these variants, but high similarity was found between the QLD, VIC/NSW-1 and VIC/NSW-3 variants with Cairns isolates (94–97%) and the NT variant was 99% and 97% similar to LSV5 and LSV3 sequences. Phylogenetic analysis of the RNA-dependent RNA polymerase (RdRp) gene showed several of the variant LSV isolates formed a separate clade related to LSV1 isolates. We propose that this clade be consolidated with related LSV strains (e.g. LSV3, LSV5) and designated LSV3. The WA-2 and VIC/NSW-2 variant LSVs were distinct from all currently known LSV strains and 86% similar to each other. We propose these variants be designated to a new LSV group, LSV8.

## Discussion

This study provides the first comprehensive molecular-based analysis of Australia’s honeybee viral landscape and highlighted key contrasts with overseas landscapes affected by *V. destructor*. We found five honeybee viruses (BQCV, LSV1, SBV, IAPV and LSV2) that were common and, in contrast to our hypothesis, virus prevalence was still high despite the absence of *V. destructor*. Virus prevalence in this study was comparable with, or higher, than similar studies in Europe^11, 45–51^, North America^33, 52, 53^ and Asia^54–56^. Differences in study design make direct comparisons difficult, but our data support that viruses can achieve high prevalence without *V. destructor* acting as a mechanical and biological vector. It is well established that honeybee viruses can persist as covert infections in colonies and high apiary level prevalence does not necessarily equate to high infection levels within hives^57^, but using NGS data as an indication of abundance suggests all five viruses were present at levels greater than expected for covert infections. It is uncertain how indicative these levels are of pre-Varroa levels or if other pathogens have induced high virus prevalence in Australia. The microsporidian parasites *Nosema apis* and *N. ceranae* have reported synergistic relationships with BQCV and potentially other viruses and could be driving the high virus prevalence in Australia^58, 59^. Interestingly BQCV in this study was highest in the winter samples (August), which coincides with peak *Nosema* spp. infections^60^, and the winter BQCV isolates formed a distinct clade. Seasonality will certainly influence virus prevalence, such that longitudinal monitoring of viruses in Australia would further add to our understanding of viral dynamics in the absence of Varroa and DWV.

We have provided strong evidence that several viruses are not present in Australian honeybees. Of particular interest is the apparent absence of the DWV complex, which is suggested to exist at low levels in all honeybee populations without *V. destructor* ^14^. While it is fundamentally difficult to prove complete absence of DWV, we have taken a weight of evidence approach that is consistent with the World Organisation for Animal Health (OIE) guidelines to support this conclusion. This approach not only considers the negative detection of DWV in our structured RT-PCR survey, but also includes the results of our sensitive NGS analysis, the lack of clinical signs of DWV-associated disease, limited pathways for virus introduction and the previous lack of detection in imported Australian bees by the USA using a NGS analysis^52^. Collectively, this evidence gives strong support for the absence of DWV in Australia. This finding contradicts two recent overseas studies that have reportedly detected DWV-A in acquired Australian bee samples^31, 32^. In both studies, short sequences were recovered by quantitative PCR from a small sample set and these showed high similarity (>98%) to local DWV-A strains. Based on our results we believe these detections were likely false positives or contamination. It seems improbable for DWV to be detected in such limited samples and not detected in this wider study using equally sensitive methods. However, our NGS analysis did map several short sequence fragments (<200 nucleotides) to the DWV reference strains under low stringency conditions. While some of these fragments were within the accepted variation of the DWV complex (up to 20%), these sequences overall were significantly different (69–74%) to each DWV strain (Supplementary material 2 and 3). Therefore it is most likely that these short sequences correspond to an undescribed distinct virus (or viruses) that is only distantly related to DWV.

Not detecting DWV in Australian *A. mellifera* also presents alternative evidence that DWV may have been absent from other populations before the arrival of *V. destructor*. Martin *et al*.^14^ demonstrated how the arrival of *V. destructor* on some Hawaiian islands and not others had resulted in the predominance of a single DWV strain. Their study is cited as evidence that DWV exists at low levels in all populations. However, Hawaii is not a completely closed population from the United States mainland and it is possible the *V. destructor*-free islands have had DWV introduced more recently through imported queens. A similar scenario may explain the presence of DWV on the *V. destructor*-free island of Newfoundland, Canada^61^. Despite import regulations, DWV may have been introduced through imported queens. There is also the possibility of contamination as samples were transferred to the United States for virus testing. Mondet *et al*.^24^ also provided an excellent study of viral dynamics in New Zealand at the *V. destructor* expansion front to show the emergence of DWV several years after the mite established. Again, it was uncertain whether DWV was present in New Zealand prior to the arrival of mites. Mondet *et al*.^24^ did not detect DWV beyond the expansion front and earlier virus testing did not detect DWV in New Zealand^62, 63^, suggesting a possible post-*V. destructor* introduction of DWV. Australia clearly provides an excellent environment to further examine the viral landscape before the potential arrival of *V. destructor*, which has been difficult to fully achieve elsewhere^64^. Although there have been some potential pathways for DWV introduction into Australia, e.g. imported queens, pest incursions, our results suggest that this virus complex has not established.

Research into the causes of bee declines observed in Europe and the USA illustrate that this is a complex problem with individual studies often leading to different conclusions. The current consensus is increased colony losses experienced in some countries are the result of multiple, interacting stresses^3, 65^. As it is very difficult to replicate these complex interactions experimentally, there is an important role for “natural experiments” and scenarios where only some of the identified potential causes of colony losses are present. Our study has confirmed that Australia’s *A. mellifera* population does not have either *V. destructor* or DWV, but it does harbour a high prevalence of other honeybee viruses as well as common fungal and bacterial diseases. In addition, Australian beekeepers have not reported repeated problems with increased colony losses of the scale seen in the Northern Hemisphere, despite being exposed to many of the same environmental stressors found overseas. Therefore, the Australian population offers a unique stage for comparative studies that can start to uncouple the importance of other stressors.

**Figure 4:**
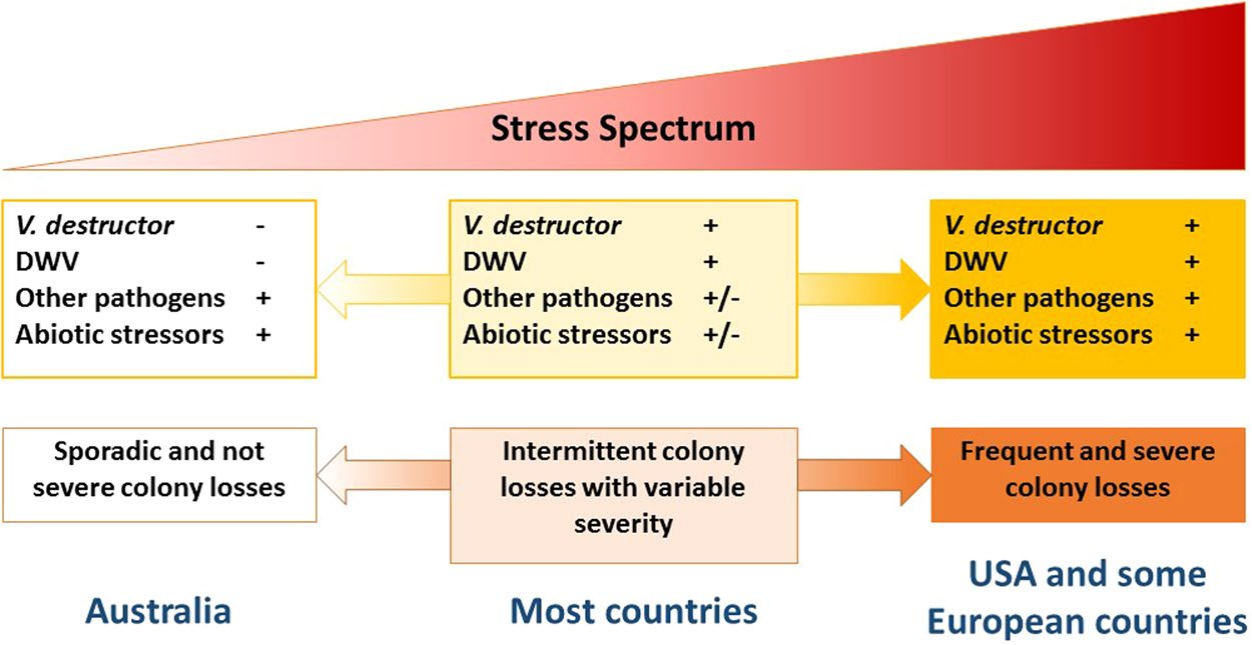
A conceptualization of *A. mellifera* colony stress as a spectrum with the current Australian situation being at one end and experiencing only sporadic colony losses and the increased colony losses of the USA and parts of Europe at the right-most extent of the spectrum. We hypothesize that a key underlying driver creating this spectrum may be *V. destructor* and DWV acting as co-pathogens, and interacting with other pathogens and abiotic stressors. Most countries are situated in the middle, with variable occurrence and severity of colony losses.

To fully interpret the Australian situation in the international context, and to graphically summarise the studies we compiled as part of our review (Supplementary material 4), we propose that colony stress might best be represented as a “spectrum” of severity, with Australia at one extreme with only sporadic colony losses and the other extreme being severe and repeated colony losses as reported in the USA (Fig. 4). We acknowledge that colony losses are not always unambiguous or reported in an unbiased or systematic way. Nonetheless, using this spectrum concept, we hypothesise that the co-pathogenic role of *V. destructor* and DWV is likely the primary underlying driver that interacts with variable environmental stressors, particularly other pathogens, agrochemicals and nutritional stress. The role of *V. destructor* and DWV as a key component in adversely affecting *A. mellifera* colony health is well established^8, 10, 11^. However, the emergence of increased colony losses in the Northern Hemisphere has led to other potential stressors such as neonicotinoid insecticides and novel pathogens to draw focus. Our analysis of Australian *A. mellifera* colonies shows that the presence of viruses other than DWV, even at high levels and combined with environmental stressors, appears to not have created increased colony losses. Therefore, we suggest that *V. destructor* and DWV is likely still the primary interaction. We note that other authors have made similar conclusions based on analysing the effect of *V. destructor* on colony health in Europe^17^, and thus our hypothesis is not entirely novel. However, by delivering new data from a *V. destructor*-free landscape, we have provided insight to enable a re-focus to the *V. destructor* – DWV co-pathogenic interaction.

Our study has also given the first direct confirmation of IAPV in Australia after being detected in the USA in imported Australian bees and being linked with Colony Collapse Disorder (CCD)^52^. High read abundance in Australian *A. mellifera* also suggests potential impacts on hive health, but in contrast there are no reports of increased colony losses or CCD. IAPV has now been shown to form a virus complex with KBV and ABPV^66^ and so earlier serological detections of KBV in Australia may have been IAPV^67^. If so, then IAPV is most likely a serotype of KBV, as Bailey *et al*.^68^ detected several serotypes of KBV in Australia. This idea is also supported by the absence of KBV in this study, whereas it was commonly detected in previous serological-based Australian surveys. In addition, IAPV was largely restricted to eastern Australia, a very similar distribution to that of KBV in previous serological-based surveys, which suggests either a more recent history for IAPV in Australia or that IAPV was previously regarded a serotype of KBV. This confusion highlights the issue of molecular-based virus identification without reference to previous identities based on serology. Interestingly, the presence of IAPV (and most likely KBV) and absence of ABPV in this population also supports a theory that these viruses occupy the same ecological niche, which leads to one virus dominating under different conditions^69^. For instance ABPV dominates in Europe, while IAPV dominates in Asia, Australia and the USA and KBV dominates in New Zealand. IAPV is still considered a serious pathogen of honeybees because of its high virulence, its occasional cause of natural honey bee mortality without an apparent synergist, and evidence that *V. destructor* is an effective biological vector^70^. However, the comparable prevalence of IAPV in Australia with overseas studies supports the hypothesis that, as a fast replicating virus, IAPV is not closely associated with *V. destructor* pathogenicity. Any *A. mellifera* brood killed by IAPV vectored by *V. destructor*, will die before emergence, thus preventing *V. destructor* offspring from leaving the capped brood cell^18^. Therefore, while *V. destructor* is capable of vectoring IAPV, the prevalence and abundance of this virus is most likely influenced more by other factors.

LSV1 and LSV2 are also new detections for Australia and extend the distribution of this virus complex. These viruses were identified as common viruses in the United States in 2009 and since then a diversity of LSV strains have been identified in Europe and North America^33, 40, 41^. With the detection of diverse Australian LSV strains it is apparent these viruses have had a long association with *A. mellifera* globally. In fact, a LSV variant was identified in a closed *A. mellifera* population on Norfolk Island, which has only had introductions of Australian honeybee stock^44^. In this study, LSV1 and LSV2 had consistently lower read abundance than the other detected honeybee viruses, suggesting a more stable coexistence with *A. mellifera*. In contrast, LSV2 has been recorded as the most abundant virus in US studies and associated with weak and collapsed colonies^41, 42^. There is much to learn about this diverse group and further characterisation of LSV variants will help focus efforts on strains relevant to honeybee health. Furthermore, if LSV1 and LSV2 have long existed in the global honeybee population, it is surprising they have only recently been detected. Perhaps both viruses were identified previously using serological methods, but were renamed after being molecularly identified, as appears to be the case with IAPV and KBV. It has been noted that bee virus X (BVX) and bee virus Y (BVY), share multiple similarities with LSV1 and LSV2 but have not yet been genetically characterised^71^. Further comparison of the physical characteristics of LSV1 and LSV2 with BVX and BVY is warranted.

This study has provided valuable insight into the honeybee viral landscape in the absence of *V. destructor* and delivered the first molecular analysis of honeybee viruses in Australia. It is clear from these findings that viruses can occur at high prevalence and abundance without the assistance of *V. destructor* mites and there is considerable viral diversity in this population. The absence of several viruses, DWV and SBPV in particular, is valuable biosecurity knowledge for Australia but additionally provides a unique opportunity for future comparative studies. Furthermore, it leads on to a testable hypothesis that *V. destructor* acting as a co-pathogen with DWV and interacting with environmental stressors is most likely to be the underlying driver of increased colony losses. Further examination and monitoring of the unique Australian *A. mellifera* population should aid our understanding of viral dynamics and pathogen interactions without the confounding effects of *V. destructor* or DWV and will contribute to global efforts to improve honeybee health.

## Methods

### Sampling strategy and collection

One-off samples from 155 apiaries totaling 1,240 hives were collected between August 2013 and April 2015 from five distinct regions in Australia (Fig. 1). Region 1 included Queensland (QLD), New South Wales (NSW), Victoria (VIC) and South Australia (SA), Region 2 was south Western Australia (WA), Region 3 was Tasmania (TAS), Region 4 was the Northern Territory (NT) and Kununurra (KUN) in north WA and Region 5 was Kangaroo Island (KI) off the SA coast. Eight seemingly healthy hives were randomly sampled at each apiary visited with two brood frames inspected from each hive to collect suspected diseased brood. Approximately 25 adult bees per hive were collected from the brood comb. An additional 124 brood samples suspected of viral disease (i.e. visually unhealthy but symptoms not consistent with bacterial or fungal disease) were collected separately to specifically test for DWV, SBPV and SBV. All samples were collected on ice, transported frozen to the laboratory and then stored at −20 °C.

### RT-PCR analysis for honeybee viruses

Adult bee samples was split into four sub-samples of 50 bees and homogenized in 5 mL of 0.05 M potassium phosphate buffer. 500 μl of homogenate was cleared by adding 50 μl of diethyl ether and 100 μl of chloroform, vigorously shaking for 30 seconds and then centrifuging for 2 minutes at 7,000 *g*. The supernatant was collected and RNA extracted using the Purelink viral RNA extraction kit (Invitrogen) and cDNA generated using the Tetro cDNA synthesis kit (Bioline) following the manufacturer’s protocol using both random hexamer (50 ng/μl) and oligo (dT)_18_ (270 ng/μl) primers and RT incubation at 40 °C for 40 min. Each sample was tested for 10 honeybee viruses; sacbrood virus (SBV), black queen cell virus (BQCV), Kashmir bee virus (KBV), Israeli acute paralysis virus (IAPV), acute bee paralysis virus (ABPV), chronic bee paralysis virus (CBPV), deformed wing virus (DWV-A and DWV-B), slow bee paralysis virus (SBPV), Lake Sinai virus 1 (LSV1) and Lake Sinai virus 2 (LSV2). PCR assays were carried out in 10 μl reactions containing 1 x PCR buffer, 1.5 mM MgCl_2_, 0.2 mM dNTPs, 0.4 μM forward primer, 0.4 μM reverse primer, 1 U Taq DNA polymerase (New England Biolabs) and 1 μl cDNA template. PCR cycling conditions were 30 cycles of 95 °C (15 s), 56 °C or 58 °C (30 sec), 72 °C (40 s) and primers are given in Supplementary material 1. PCR reactions were analysed on 1.2% agarose gels stained with GelRed (Jomar Biosciences) and all positive PCR products were confirmed by Sanger sequencing.

### RNA preparation and library construction for NGS

Nine pooled adult bee samples from the nine sampling periods (VIC/NSW-1 – August 2013, VIC/NSW-2 – April 2014, VIC/NSW-3 – August 2014, SA – August 2014, QLD – October 2014, WA-1 – October 2013, WA-2 – October 2014, TAS – November 2014, NT – June 2014) were chosen for NGS. Pooled samples were created from 1 ml of 50 homogenized bees per apiary sample into a screw-cap centrifuge tube and adjusting with 0.05 M potassium phosphate buffer for a total volume of 20 ml. 3 ml diethyl ether and 3 ml chloroform were added, shaken vigorously and centrifuged at 6,000 rpm for 30 minutes (J-E Avanti centrifuge). Supernatants were transferred to Ultraclear SW28 tubes (Beckman Coultier) and centrifuged at 21,500 rpm for 3.5 hours at 4 °C (Beckman L-80 ultracentrifuge). Pelleted samples were dissolved in 1 ml of 0.05 M potassium phosphate buffer then passed through a 0.22 μm filter to remove bacterial contamination. We then mixed 340 μl of each filtered sample with 10 μl of RNase, 10 μl DNase and 40 μl of DNase I buffer and incubated at 37 °C for 2 hours. RNA was extracted from the treated samples using the Purelink viral RNA extraction kit. Illumina libraries were prepared using the TruSeq stranded mRNA library prep kit (Illumina, San Diego, CA) and 100 bp paired-end sequences were generated on an Illumina HiSeq. 2500 run in rapid mode at the Biomolecular Research Facility (Australian National University, Canberra). An average of 93 million paired-end reads were generated per library. Bioinformatics analysis of RNA-seq data was carried out with the CLC Genomic workbench (Qiagen, Aarhus). Raw data were quality trimmed and adapter sequences removed before the trimmed reads were mapped to reference virus genomes from the NCBI GenBank database of the honeybee viruses tested for by RT-PCR. Mapping parameters were first set with a length fraction of 0.9 and similarity fraction of 0.9, and a second mapping was run with the similarity fraction set to 0.7. Consensus sequences were manually inspected for genome coverage and similarity to mapped reference genomes using BLASTn. Trimmed data were also *de novo* assembled in CLC Genomics workbench using default parameters and a minimum contig length of 1,000 nucleotides. Contigs were compared against the NCBI non-redundant protein database using BLASTx and contigs of interest were inspected manually to detect potential variant genomes. Phylogenetic analysis of viral genomes were done in MEGA7^72^. Mulitple amino acid sequence alignments with relevant reference sequences were performed with MUSCLE and consensus maximum-likelihood phylogenetic trees were constructed using appropriate models for each virus and 1,000 bootstraps.

### Semi-systematic review of international bee virus studies

To place our results within the international context and the current debates on the causes of increased honeybee colony losses, we undertook a semi-systematic review of the literature to find comparable virus studies. Studies that presented prevalence data for multiple viruses across large areas or in relation to colony losses were included. The results from these studies were extracted into a summary table (Supplementary material 4).

## Electronic supplementary material

Supplementary material
