# Supplementary material for: Absence of deformed wing virus and Varroa destructor in Australia provides unique perspectives on honeybee viral landscapes and colony losses
Source: Sci Rep. 2017 Jul 31;7:6925. doi: 10.1038/s41598-017-07290-w (PMC5537221; doi:10.1038/s41598-017-07290-w)
Supplement: Supplementary file 1 — Supplementary material [file 41598_2017_7290_MOESM1_ESM.pdf]

Absence of deformed wing virus and *Varroa destructor* in Australia provides unique perspectives on honeybee viral landscapes and colony losses

John M K Roberts[1*], Denis L Anderson[1,2] and Peter A Durr[3]

[1]Commonwealth Scientific and Industrial Research Organisation, Canberra, ACT, 2601, Australia

[2] ADFCA, Research and Development Division, Al Ain, UAE

[3] CSIRO-Australian Animal Health Laboratory, Geelong, Victoria, 3219, Australia

*Author for correspondence

Supplementary material 1. Primers used for RT-PCR virus detection. T_a is the PCR annealing temperature.

Virus	Primers	T_a	Reference
IAPV	F: AGACACCAATCACGGACCTCAC	58 °C	Maori et al 2007[1]
	R: AGATTTGTCTGTCTCCCAGTGCACAT		
KBV	F: GATGAACGTCGACTATTGA	58 °C	Stoltz et al 1995[2]
	R: TGTGGGTTGGCTATGAGTCA		
BQCV	F: TGG TCA GCT CCC ACT ACC TAA AAC	58 °C	Benjeddou et al 2001[3]
	R: GCA ACA AGA AAC GTA AAC CAC		
LSV1	F: TTA TCT CGC GCC GCC ACC TC	52 °C	Runckel et al 2011[4]
	R: ATC GCC GCT GCA ACG TGA CC		
LSV2	F: CGG CCG GTC TAG CGT GGT TG	52 °C	Runckel et al 2011[4]
	R: TGG CAA GCT GTG ACG AAT CCC T		
DWV-A/	F: ATA TTC ACG GAT TGT TTG AAA GA	58 °C	Meeus et al 2010[5]
DWV-B	R: CRC TAA CAT TCA TGA TAA GAT CGT C		
SBPV	F: GAT TTG CGG AAT CGT AAT ATT GTT TG	58 °C	de Miranda 2010[6]
	R: ACC AGT TAG TAC ACT CCT GGT AAC TTC G		
SBV	F: AAT GGT GCG GTG GAC TAT GG	58 °C	Grabensteiner et al 2001[7]
	R: TGA TAC AGA GCG GCT CGA CA		
ABPV	F: TGA GAA CAC CTG TAA TGT GG	58 °C	Tentcheva et al 2004[8]
	R: ACC AGA GGG TTG ACT GTG TG		
CBPV	F: AGT TGT CAT GGT TAA CAG GAT ACG AG	58 °C	Ribiere et al 2000[9]
	R: TCT AAT CTT AGC ACG AAA GCC GAG		

Supplementary material 2. Summary of BLAST results for sequence fragments from NT, QLD and WA-2 samples that mapped to DWV reference genomes.

**Sequence fragment and length**	**BLAST aligned sequence**	**Location on aligned sequence**	**Highest nucleotide similarity to DWV isolates**
NT DWV-like 1 (54bp)	GTAGGGACCCCTCTATCCTCTAGGTACTGTATGTCGCGAAAGTATGAAAGTAAT	734-787	91% similar to DWV isolate leuven-dwv1 (KX783225)
NT DWV-like 2 (127bp)	ATATGCGATGTACCTAATACAATACCTTTCAAGGTGCATGCATATTGGCGTGGAGAAATGCAAGTTCGAATACAAGTTAACTCGAATAAGTTCCAGGTTGGTCAATTGC AAGCCACTTGGTACTATT	2045-2171	84% similar to DWV isolate leuven-dwv1 (KX783225)
NT DWV-like 3 (66bp)	TGTTTATGTATGTGCAAGTACCATTGATACCGATGGAAGCTGTAGCTGATACTATCGATATTAATG	3189-3254	92% similar to DWV isolate leuven-dwv1 (KX783225)
NT DWV-like 4 (148bp)	TGAAATGATGAATACTGTCATCACCATCGTAAAACGATTGTTAGGCAAATATAGATTGGCGACGCAACCCCAGGACCACGCCGATTCACGTACTGTTAACGCTACCCCA GAGGGACCTAGTGCTGAGGCTGAGGAAACAAGTGCGTGG	4867-5017	75% similar to DWV isolate leuven-dwv1 (KX783225)
NT DWV-like 5 (51bp)	GTGAAGATGAACCTGAAATTGTAAAGCGATGGGTTAAAGAATGTTTGTATT	5240-5291	89% similar to DWV isolate leuven-dwv1 (KX783225)
NT DWV-like 6 (421bp)	ATTTTGTTGCATGACCTTACAGCTGAGATGAATCAGTCGAGAAATTTGACTGTGTTTACTCGGGTGTATGATCAAATATCGAAATTGAAGACCGACCTGAATGTTTTAC GATTTGTATGACTGGTGCTCCTGGTATTGGTAAGTCATTCTTAACTGATGCAAGTAGTACTCCCGTAACTACGGGCATCAAGTGTGTCGTTAATCCTTTATCAGATTATT GGGATCAGTGTGACTTTCAACCAGTCTTGTGCGTAGACGACATGTGGAGTGTTGAGACTGGAGCAACTTTGGACAAACAACTAAATTTGCTCTTTCAGGTTCATTCTCC AATTGTCCTAAAGCTGATTTAGATGGTAAGAAGATGAGGTATAATCCTGAAATCTTCATTTATAATACCAATAAACCTTTTCCGGTATTTGAT	5375-5863	72% similar to DWV isolate leuven-dwv1 (KX783225)
NT DWV-like 7 (95bp)	TTTGATAATCCTACGTGGCGATTAATATATAATGCATCAAGAAAGGGTTTGCCAGAATATTATACACTACCAAGTGATGAAATTTCATTAGATTC	7049-7143	78% similar to DWV isolate leuven-dwv1 (KX783225)
NT DWV-like 8 (203bp)	CACTTGAAAGAGAAGTTTATAACCGTTATTAAACCTATAAACGGTTGTAAAATTCGTAGTCTGCAGGATGCTATTTTTGGCGCACCTGGAGTAGAAGGATATGATTCGA TTTCGTGGAATACGAGTGCCGGTTTTCCGTTGTCTACAATGAAGCCGGCTGGAACATCAGGAAAGCGCTGGTTGTTTGATATTGAACTTACAAG	8480-8681	77% similar to DWV isolate leuven-dwv1 (KX783225)
NT DWV-like 9 (153bp)	GGGCATAAAGCCACATACGATATTTACTGACTGCCTCAAAGATACATGTTTGCCCATAGAGAAGTGTATGATACCGGGTAAAACGAGGATATTTAGTATTGCGCCTGTT CAATATACAATACCTTTTCGACAATATTTTCTCGATTTTATGGC	8761-8913	77% similar to DWV isolate leuven-dwv1 (KX783225)
NT DWV-like 10	ATTATACAGAGGAAAAAGATAAGACTGAGATGAGTCGAGTCATGTGGACTATGGCTCAGGAGATTCTTGCACCAAGCCATCTTTGTAGAGATTTGGTGTATAGAGTAC	9111-9253	75% similar to DWV isolate leuven-dwv1 (KX783225)

(143bp)	CTTGTGGCATACCTTCGGGCTCACCCATCACGGAC		
NT DWV-like 11 (72bp)	CGTGTAGCCACTATAGAAAATGCTAAACAATCTCTAGAGTTGGCATTCGGTTGGGGTCCGCAATACTTCAAT	9626-9697	83% similar to DWV isolate leuven-dwv1 (KX783225)
NT DWV-like 12 (44bp)	AAGCTTGGTATTTATGAGGATCTCATTACATGGGAGGAGATGGA	9731-9774	89% similar to DWV isolate leuven-dwv1 (KX783225)
QLD DWV-like 1 (105bp)	TAGACCTCTACGGCATCGAGTAGAGATTAAAAAGAGTAGTGCAATAGTATAATCACTGTCTACCACCACATATGGTAATGATATGGTTTGTGGTAAACCGGTTAT	488-592	80% similar to VDV-1 genome (AY251269)
QLD DWV-like 2 (99bp)	ATGTCAGGTTATATTATGAATGCTCGAGTATGATTTTCTGCGTTGGAGTCGGGACCCCTCGGTCTCCCAGGTGTCGCATGTGACGAAAGTGCGAAAATA	687-785	80% similar to VDV-1 genome (AY251269)
QLD DWV-like 3 (135bp)	ATATGCGATGTACCTAATACAATACCATTCAAGGTACATGCATATTGGCGTGGAGAGATGCAAGTTCGAATACAAGTTAATTCGAATAAATTCCAAGTAGGTCAATTGCAAGCCACTTGGTATTATATGGATTAT	2045-2179	84% similar to VDV-1 genome (AY251269)
QLD DWV-like 4 (232bp)	ATGGATTATATGACACTTAAAGCATCAAGTTATGTGGTATTTGATTTGCAAGAAACAAATAGCTTTACGTTTGAAGTGCCTTATGTGTCATATAGACCATGGTGGTGTTTATGTATGTACAAGTACCGTTGATACCTATGGAAGCTGTTGCAGATACCATAGATATCAATGTGTATTTAAGAGGAGGCTCTTCATTTGAAGTATGTATTCCTGTACAACCTAGTGTAGGATT	3029-3315	69% similar to VDV-1 genome (AY251269)
QLD DWV-like 5 (326bp)	TTTCCGCAACACCAGAAGGACCTAGTGCTGAGGCTGAGGAAACAAGTGCGTGGGTATCCGTTATTTATAACGGTGTTTGTAATATGTTGAATGTGGTGGCACAAAAGCCAAAACAATTTAAAGACTGGGTAAAAATTGTTTCCCAAGATTTTGGAAACAATTGTAGAAGTAGTAATCAGGTATTCATATTCTTTAAGAACACCTTTGAGGTTTTGAAGAATTTGTAGGACTAATCCTGCCGCTCGCCTATTGAAGGCTGTGAGCGATGAGCCCGAGATACTCAAAGCGTGGGTTAAAGAATGTTTGTACTTAAATGATCCCAAATT	4965-5307	77% similar to VDV-1 genome (AY251269)
QLD DWV-like 6 (111bp)	TACACCTGTCACCACTGGAATTAAGTGTGTTGTTAATCCCCTCTCTGACTATTGGGATCAGTGTGATTTCCAACCGGTTTTGTGCGTTGATGATATGTGGAGTGTGGAAAC	5599-5709	82% similar to VDV-1 genome (AY251269)
QLD DWV-like 7 (98bp)	AACTATTTTAGTTATTTATTCCATAAATCATGGTTATACGCAAATCCCACGTGGAGACTGATTTTTAATGGTTCAAAGAAAGGTATGCCAGAGTATTT	7013-7110	80% similar to VDV-1 genome (AY251269)
QLD DWV-like 8 (90bp)	TGGAATACGAGTGCAGGTTTTCCATTGTCAACAATGAAACCTGCTGGAACATCTGGAAAGAGATGGTTGTTTGATATTGAACTAAAAGAT	8594-8683	80% similar to VDV-1 genome (AY251269)
QLD DWV-like 9 (88bp)	CAACGACTCAAGAAATGAGAAAGAGAGGAATCAAGCCTCACACCATCTTCACAGATTGCCTCAAAGATACATGTTTGCCAGTAGAAAA	8736-8823	82% similar to VDV-1 genome (AY251269)
QLD DWV-like 10	AGGGAAGTACCACCCCCCCACTCGTTCGTTTTTAAATCTGCTAATAGGAATGAACTCTATTATAAGAGTCCAAAAGCAGTGTGGATTAGACCATCACTTTTGGCTTATAC	9854-10050	85% similar to VDV-1 genome (AY251269)

(198bp)	TAGAGAAAGGATGAGCTGCCTCTAAAGGCTCAATTCCGTAGTAGAGTAGGTTTAATTAGGATTAAAGTGGTACTCTAGGTTAGGTATT		
WA-2 DWV-like 1 (127bp)	ATAGGTAATGATCCCAATACGGTACCATTTAGAGTACATGCATATTGGCGTGGAGATATTGAAGTTAAAATTCAGATAAATTCTAATAAATTTCAAGTTGGGCAATTGC AAGCTACTTGGTATTATT	2072-2198	84% similar to DWV isolate Chilensis A1 (JQ413340)
WA-2 DWV-like 2 (91bp)	TGGCGTGGATCTTTGGAGTATCGATTTGATATCGTTGCTTCTCAATTTCATACTGGTAGGTTGATTGTAGGCTATATCCCCGGATATGACA	2948-3037	80% similar to DWV isolate Chilensis A1 (JQ413340)
WA-2 DWV-like 3 (98bp)	AATTGTCAAAGTAGTAATCAAGTATTTATTTTCTTTAAGAATACATTTGAAGTATTAAAGAAGACTTGGGCTTATTTATTTTGTCGAAGTAATCCTGC	5150-5247	81% similar to DWV isolate Chilensis A1 (JQ413340)

Supplementary material 3. Alignment of mapped reads from the NT sample to DWV-A (NC_004830), DWV-B (NC_006494) and DWV-C (ERS657949) using Geneious (Version 9.1.5, created by Biomatters, Auckland, New Zealand)

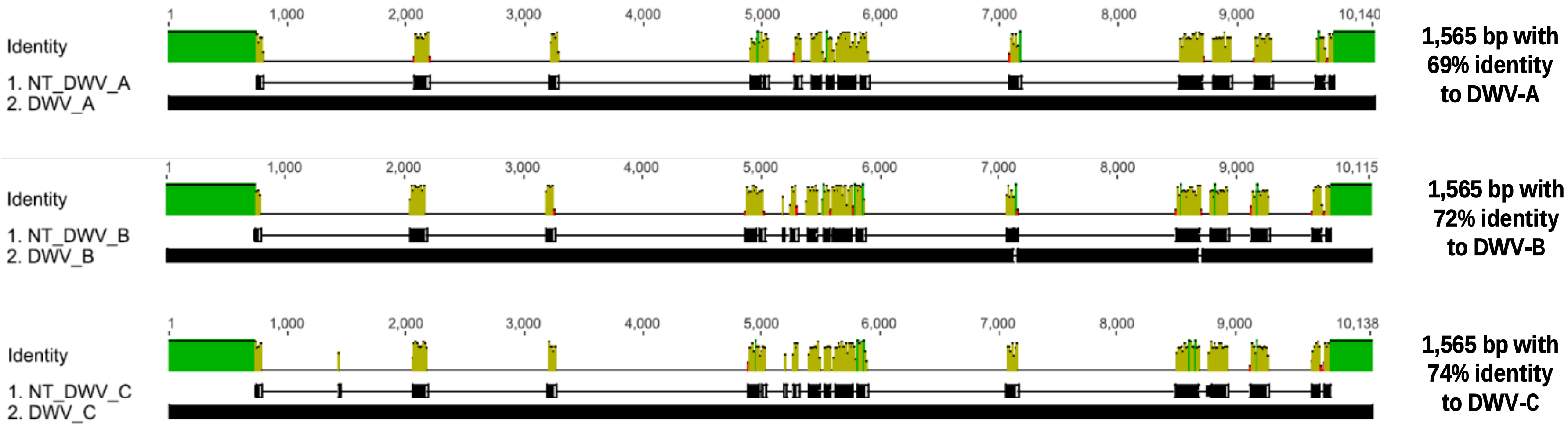

Supplementary material 4. Australian apiary survey results within the context of comparable molecular-based surveys. In relation to Figure 4, populations with profiles 1-3 are on the lower end of the stress spectrum, populations with profile 4 range across the middle of the stress spectrum and populations with profile 5 are on the high end of the stress spectrum.

**Survey reference**	**Country: Region**	**Increased colony losses recorded**	**Colony stressors**						**Stress profile**	**Commercial-scale beekeepers**
			Varroa destructor	**DWV complex**	**Other viruses detected**	**Viruses tested for but not detected**	**Other pathogens**	**Abiotic stressors**		
This survey	Australia	No	No	No	BQCV<br>SBV<br>LSV1<br>LSV2<br>IAPV<br>ALPV	DWV<br>SBPV<br>KBV<br>CBPV<br>ABPV	Most	Most	1	Yes
Malfroy et al. [10]	Australia: Norfolk Island	No	No	No	LSV	DWV<br>ABPV<br>IAPV<br>KBV<br>BQCV<br>SBV<br>CBPV<br>SBPV	Few	Few	2	No

Kajobe et al. [11]	Uganda	No	No	No	BQCV	DWV<br>SBV<br>CBPV<br>ABPV<br>IAPV	Few	Few	2	No
Shutler et al. [12]	Canada : Newfoundland	No	No	DWV-A	BQCV	IAPV<br>KBV<br>SBV	Few	Few	3	No
Sanpa and Chantawannakul [13]	Thailand : Northern provinces	No	Yes	DWV-A	ABPV<br>KBV<br>SBV	BQCV<br>CBPV	Most	Some	4	Yes
Ai et al. [14]	China	No	Yes	DWV-A	ABPV<br>IAPV<br>BQCV<br>SBV<br>CBPV	KBV	Most	Most	4	Yes
Kojima et al. [15]	Japan	No	Yes	DWV-A	BQCV<br>SBV<br>IAPV	CBPV<br>KBV<br>ABPV	Most	Most	4	Yes
Strauss et al. [16]	South Africa	No	Yes	DWV-B	BQCV<br>IAPV	DWV-A<br>SBV<br>CBPV<br>KBV<br>VdMLV	Most	Some	4	Yes
Muli et al. [17]	Kenya	No	Yes	DWV-A	BQCV<br>ABPV	SBV<br>KBV<br>IAPV	Some	Some	4	No

						CBPV				
Yang et al. [18]	China	No	Yes	DWV-A	BQCV<br>IAPV<br>SBV<br>CBPV	ABPV<br>KBV	Most	Most	4	Yes
Mondet et al. [19]	New Zealand	No	Yes	DWV-A	BQCV<br>KBV<br>SBV<br>CBPV		Some	Most	4	Yes
Adjlane et al. [20]	Algeria	No	Yes	DWV-A	BQCV<br>SBV<br>ABPV<br>IAPV<br>CBPV	DWV-B	Most	Some	4	No
Tsevegmid et al. [21]	Mongolia: northern regions	No	Yes	DWV-A	SBV<br>BQCV<br>CBPV	ABPV<br>KBV<br>IAPV<br>LSV2	Some	Some	4	No
Giacobino et al. [22]	Argentina	No	Yes	DWV-A	BQCV<br>ABPV<br>CBPV	KBV<br>IAPV	Most	Some	4	Yes
Anido et al. [23]	Uruguay	No	Yes	DWV-A	BQCV<br>SBV<br>ABPV	IAPV<br>KBV	Most	Some	4	Yes
Teixeira et al. [24]	Brazil: southeast	No	Yes	DWV-A	BQCV<br>ABPV	KBV<br>CBPV	Most	Some	4	Yes

	region					SBV				
Soroker et al. [25]	Israel	No	Yes	DWV-A<br>DWV-B	BQCV<br>SBV<br>ABPV<br>IAPV	KBV<br>CBPV	Most	Most	4	Yes
Rodriguez et al. [26]	Chile: Biobio region	No	Yes	DWV-A	BQCV<br>SBV<br>ABPV		Most	Some	4	Yes
Desai et al. [27]	Canada	Yes	Yes	DWV-A	BQCV<br>SBV<br>ABPV<br>KBV<br>IAPV<br>CBPV		Most	Most	5	Yes
Forgach et al. [28]	Hungary	Yes	Yes	DWV-A	BQCV<br>SBV<br>ABPV	KBV<br>CBPV	Most	Most	5	Yes
Tentcheva et al. [8]	France	Yes	Yes	DWV-A	BQCV<br>SBV<br>ABPV<br>KBV<br>CBPV		Most	Most	5	Yes
Berenyi et al. [29]	Austria	Yes	Yes	DWV-A	BQCV<br>SBV<br>ABPV<br>CBPV	KBV	Most	Most	5	Yes

Genersch et al. [30]	Germany	Yes	Yes	DWV-A	SBV<br>ABPV<br>KBV	IAPV	Most	Most	5	Yes
Berthoud et al. [31]	Switzerland	Yes	Yes	DWV-A	ABPV	KBV<br>CBPV	Most	Most	5	Yes
Nguyen et al. [32]	Belgium	Yes	Yes	DWV-A	BQCV<br>SBV<br>ABPV<br>CBPV		Most	Most	5	Yes
Baker and Schroeder [33]	England: Devon	Yes	Yes	DWV-A	SBV<br>BQCV<br>ABPV	KBV<br>CBPV	Most	Most	5	Yes
Antunez et al. [34]	Spain	Yes	Yes	DWV-A	BQCV<br>SBV<br>IAPV<br>KBV		Most	Most	5	Yes
Cornman et al. [35]	USA	Yes	Yes	DWV-A	BQCV<br>SBV<br>LSV1<br>LSV2<br>ABPV<br>IAPV<br>KBV		Most	Most	5	Yes
Cox-Foster et al. [36]	USA	Yes	Yes	DWV-A	CBPV<br>SBV<br>BQCV<br>ABPV		Most	Most	5	Yes

					KBV<br>IAPV					
Dainat et al. [37]	Switzerland	Yes	Yes	DWV-A	BQCV<br>SBV<br>SBPV<br>ABPV	IAPV<br>KBV<br>CBPV	Most	Most	5	Yes
Budge et al. [38]	England and Wales	Yes	Yes	DWV-A	BQCV<br>CBPV<br>SBV ABPV<br>KBV		Most	Most	5	Yes
Welch et al. [39]	USA: Massachusetts	Yes	Yes	DWV-A	BQCV<br>SBV	ABPV<br>KBV<br>IAPV<br>CBPV	Most	Most	5	Yes
Bacandritsos et al. [40]	Greece	Yes	Yes	DWV-A	CBPV<br>BQCV<br>SBV<br>ABPV		Most	Most	5	Yes
Runckel et al. [4]	USA	Yes	Yes	DWV-A	BQCV<br>CBPV<br>SBV<br>ABPV<br>KBV<br>IAPV		Most	Most	5	Yes
Traynor et al. [41]	USA	Yes	Yes	DWV-A	BQCV<br>SBV<br>CBPV	SBPV	Most	Most	5	Yes

					LSV2<br>ABPV<br>IAPV<br>KBV					
Tozkar et al. [42]	Turkey	Yes	Yes	DWV-A<br>DWV-B	BQCV<br>LSV<br>CBPV<br>ABPV<br>KBV<br>IAPV	SBV<br>SBPV	Most	Some	5	Yes
Ravoet et al. [43]	Belgium	Yes	Yes	DWV-A	BQCV<br>SBV CBPV<br>LSV<br>ABPV<br>VdMLV<br>ALPV	IAPV<br>SBPV	Most	Most	5	Yes
Amiri et al. [44]	Denmark	Yes	Yes	DWV-A	SBV<br>BQCV<br>CBPV<br>ABPV<br>KBV		Most	Most	5	Yes
Gajger et al. [45]	Croatia	Yes	Yes	DWV-A	BQCV<br>SBV<br>ABPV<br>CBPV	KBV<br>IAPV	Most	Most	5	Yes
Granberg et al. [46]	Spain: Navarre	Yes	Yes	No	IAPV<br>ALPV	BQCV<br>SBV<br>ABPV	Most	Most	5	Yes

					LSV	KBV<br>DWV				
Nielsen et al. [47]	Denmark	Yes	Yes	DWV-A	BQCV<br>CBPV<br>SBV<br>ABPV<br>KBV		Most	Most	5	Yes

Supplementary material references

1. Maori, E. *et al.* Isolation and characterization of Israeli acute paralysis virus, a dicistrovirus affecting honeybees in Israel: evidence for diversity due to intra- and inter-species recombination. *Journal of General Virology* **88**, 3428-3438, doi:10.1099/vir.0.83284-0 (2007).
2. Stoltz, D., Shen, X. R., Boggis, C. & Sisson, G. Molecular diagnosis of Kashmir bee virus infection. *Journal of Apicultural Research* **34**, 153-160 (1995).
3. Benjeddou, M., Leat, N., Allsopp, M. & Davison, S. Detection of acute bee paralysis virus and black queen cell virus from honeybees by reverse transcriptase PCR. *Applied and Environmental Microbiology* **67**, 2384-2387, doi:10.1128/aem.67.5.2384-2387.2001 (2001).
4. Runckel, C. *et al.* Temporal analysis of the honey bee microbiome reveals four novel viruses and seasonal prevalence of known viruses, *Nosema* and *Crithidia*. *PLOS One* **6**, e20656 (2011).
5. Meeus, I., Smagghe, G., Siede, R., Jans, K. & de Graaf, D. C. Multiplex RT-PCR with broad-range primers and an exogenous internal amplification control for the detection of honeybee viruses in bumblebees. *Journal of Invertebrate Pathology* **105**, 200-203 (2010).
6. de Miranda, J. R. *et al.* Genetic characterization of slow bee paralysis virus of the honeybee (Apis mellifera L.). *Journal of General Virology* **91**, 2524-2530, doi:10.1099/vir.0.022434-0 (2010).
7. Grabensteiner, E. *et al.* Sacbrood virus of the honeybee (Apis mellifera): Rapid identification and phylogenetic analysis using reverse transcription-PCR. *Clinical and Diagnostic Laboratory Immunology* **8**, 93-104 (2001).
8. Tentcheva, D. *et al.* Prevalence and seasonal variations of six bee viruses in Apis mellifera L. and Varroa destructor mite populations in France. *Applied and Environmental Microbiology* **70**, 7185-7191, doi:10.1128/aem.70.12.7185-7191.2004 (2004).
9. Ribiere, M., Faucon, J. P. & Pepin, M. Detection of chronic honey bee (Apis mellifera L.) paralysis virus infection: application to a field survey. *Apidologie* **31**, 567-577, doi:10.1051/apido:2000147 (2000).

10 Malfroy, S. F., Roberts, J. M. K., Perrone, S., Maynard, G. & Nadine, C. A pest and disease survey of the isolated Norfolk Island honey bee (*Apis mellifera*) populationA pest and disease survey of the isolated Norfolk Island honey bee (Apis mellifera) population. *Journal of Apicultural Research*, doi:10.1080/00218839.2016.1189676 (2016).

11 Kajobe, R. *et al.* First molecular detection of a viral pathogen in Ugandan honey bees. *Journal of Invertebrate Pathology* **104**, 153-156, doi:10.1016/j.jip.2010.02.007 (2010).

12 Shutler, D. *et al.* Honey Bee Apis mellifera Parasites in the Absence of Nosema ceranae Fungi and Varroa destructor Mites. *Plos One* **9**, doi:10.1371/journal.pone.0098599 (2014).

13 Sanpa, S. & Chantawannakul, P. Survey of six bee viruses using RT-PCR in Northern Thailand. *Journal of Invertebrate Pathology* **100**, 116-119, doi:10.1016/j.jip.2008.11.010 (2009).

14 Ai, H. X., Yan, X. & Han, R. C. Occurrence and prevalence of seven bee viruses in Apis mellifera and Apis cerana apiaries in China. *Journal of Invertebrate Pathology* **109**, 160-164, doi:10.1016/j.jip.2011.10.006 (2012).

15 Kojima, Y. *et al.* Infestation of Japanese Native Honey Bees by Tracheal Mite and Virus from Non-native European Honey Bees in Japan. *Microbial Ecology* **62**, 895-906, doi:10.1007/s00248-011-9947-z (2011).

16 Strauss, U. *et al.* Seasonal prevalence of pathogens and parasites in the savannah honeybee (Apis mellifera scutellata). *Journal of Invertebrate Pathology* **114**, 45-52, doi:10.1016/j.jip.2013.05.003 (2013).

17 Muli, E. *et al.* Evaluation of the Distribution and Impacts of Parasites, Pathogens, and Pesticides on Honey Bee (Apis mellifera) Populations in East Africa. *Plos One* **9**, doi:10.1371/journal.pone.0094459 (2014).

18 Yang, B., Peng, G., Li, T. & Kadowaki, T. Molecular and phylogenetic characterization of honey bee viruses, Nosema microsporidia, protozoan parasites, and parasitic mites in China. *Ecology and Evolution* **3**, 298-311, doi:10.1002/ece3.464 (2013).

19 Mondet, F., de Miranda, J. R., Kretzschmar, A., Le Conte, Y. & Mercer, A. R. On the Front Line: Quantitative Virus Dynamics in Honeybee (Apis mellifera L.) Colonies along a New Expansion Front of the Parasite Varroa destructor. *Plos Pathogens* **10**, doi:10.1371/journal.ppat.1004323 (2014).

20 Adjlane, N., Dainat, B., Gauthier, L. & Dietemann, V. Atypical viral and parasitic pattern in Algerian honey bee subspecies Apis mellifera intermissa and A-m. sahariensis. *Apidologie* **47**, 631-641, doi:10.1007/s13592-015-0410-x (2016).

21 Tsevegmid, K., Neumann, P. & Yanez, O. The Honey Bee Pathosphere of Mongolia: European Viruses in Central Asia. *Plos One* **11**, doi:10.1371/journal.pone.0151164 (2016).

22 Giacobino, A. *et al.* Varroa destructor and viruses association in honey bee colonies under different climatic conditions. *Environmental Microbiology Reports* **8**, 407-412, doi:10.1111/1758-2229.12410 (2016).

23 Anido, M. *et al.* Prevalence and distribution of honey bee pests and pathogens in Uruguay. *Journal of Apicultural Research* **54**, 532-540, doi:10.1080/00218839.2016.1175731 (2015).

24 Teixeira, E. W., Chen, Y. P., Message, D., Pettis, J. & Evans, J. D. Virus infections in Brazilian honey bees. *Journal of Invertebrate Pathology* **99**, 117-119, doi:10.1016/j.jip.2008.03.014 (2008).

25 Soroker, V. *et al.* Evaluation of colony losses in Israel in relation to the incidence of pathogens and pests. *Apidologie* **42**, 192-199, doi:10.1051/apido/2010047 (2011).

26 Rodriguez, M. *et al.* Prevalence and phylogenetic analysis of honey bee viruses in the Biobio Region of Chile and their association with other honey bee pathogens. *Chilean Journal of Agricultural Research* **74**, 170-177, doi:10.4067/s0718-58392014000200007 (2014).

27 Desai, S. D., Kumar, S. & Currie, R. W. Occurrence, detection, and quantification of economically important viruses in healthy and unhealthy honey bee (Hymenoptera: Apidae) colonies in Canada. *Canadian Entomologist* **148**, 22-35, doi:10.4039/tce.2015.23 (2016).

28 Forgach, P., Bakonyi, T., Tapaszti, Z., Nowotny, N. & Rusvai, M. Prevalence of pathogenic bee viruses in Hungarian apiaries: Situation before joining the European Union. *Journal of Invertebrate Pathology* **98**, 235-238, doi:10.1016/j.jip.2007.11.002 (2008).

29 Berenyi, O., Bakonyi, T., Derakhshifar, I., Koglberger, H. & Nowotny, N. Occurrence of six honeybee viruses in diseased Austrian apiaries. *Applied and Environmental Microbiology* **72**, 2414-2420, doi:10.1128/aem.72.4.2414-2420.2006 (2006).

30 Genersch, E. *et al.* The German bee monitoring project: a long term study to understand periodically high winter losses of honey bee colonies. *Apidologie* **41**, 332-352, doi:10.1051/apido/2010014 (2010).

31 Berthoud, H., Imdorf, A., Haueter, M., Radloff, S. & Neumann, P. Virus infections and winter losses of honey bee colonies (Apis mellifera). *Journal of Apicultural Research* **49**, 60-65, doi:10.3896/ibra.1.49.1.08 (2010).

32 Nguyen, B. K. *et al.* Effects of honey bee virus prevalence, Varroa destructor load and queen condition on honey bee colony survival over the winter in Belgium. *Journal of Apicultural Research* **50**, 195-202, doi:10.3896/ibra.1.50.3.03 (2011).

33 Baker, A. & Schroeder, D. Occurrence and genetic analysis of picorna-like viruses infecting worker bees of Apis mellifera L. populations in Devon, South West England. *Journal of Invertebrate Pathology* **98**, 239-242, doi:10.1016/j.jip.2008.02.010 (2008).

34 Antunez, K. *et al.* Low prevalence of honeybee viruses in Spain during 2006 and 2007. *Research in Veterinary Science* **93**, 1441-1445, doi:10.1016/j.rvsc.2012.03.006 (2012).

35 Cornman, R. S. *et al.* Pathogen webs in collapsing honey bee colonies. *Plos One* **7**, doi:10.1371/journal.pone.0043562 (2012).

36 Cox-Foster, D. L. *et al.* A metagenomic survey of microbes in honey bee colony collapse disorder. *Science* **318**, 283-287, doi:10.1126/science.1146498 (2007).

37 Dainat, B., Evans, J. D., Chen, Y. P., Gauthier, L. & Neumann, P. Predictive Markers of Honey Bee Colony Collapse. *Plos One* **7**, doi:10.1371/journal.pone.0032151 (2012).

38 Budge, G. E. *et al.* Pathogens as Predictors of Honey Bee Colony Strength in England and Wales. *Plos One* **10**, doi:10.1371/journal.pone.0133228 (2015).

39 Welch, A., Drummond, F., Tewari, S., Averill, A. & Burand, J. P. Presence and Prevalence of Viruses in Local and Migratory Honeybees (Apis mellifera) in Massachusetts. *Applied and Environmental Microbiology* **75**, 7862-7865, doi:10.1128/aem.01319-09 (2009).

40 Bacandritsos, N. *et al.* Sudden deaths and colony population decline in Greek honey bee colonies. *Journal of Invertebrate Pathology* **105**, 335-340, doi:10.1016/j.jip.2010.08.004 (2010).

41 Traynor, K. S. *et al.* Multiyear survey targeting disease incidence in US honey bees. *Apidologie* **47**, 325-347, doi:10.1007/s13592-016-0431-0 (2016).

42 Tozkar, C., Kence, M., Kence, A., Huang, Q. & Evans, J. Metatranscriptomic analyses of honey bee colonies. *Frontiers in genetics* **6**, 100 (2015).

43 Ravoet, J. *et al.* Comprehensive bee pathogen screening in Belgium reveals Crithidia mellificae as a new contributory factor to winter mortality. *Plos One* **8**, doi:10.1371/journal.pone.0072443 (2013).

44 Amiri, E., Meixner, M., Nielsen, S. L. & Kryger, P. Four Categories of Viral Infection Describe the Health Status of Honey Bee Colonies. *Plos One* **10**, doi:10.1371/journal.pone.0140272 (2015).

45 Gajger, I. T., Kolodziejek, J., Bakonyi, T. & Nowotny, N. Prevalence and distribution patterns of seven different honeybee viruses in diseased colonies: a case study from Croatia. *Apidologie* **45**, 701-706, doi:10.1007/s13592-014-0287-0 (2014).

46 Granberg, F. *et al.* Metagenomic Detection of Viral Pathogens in Spanish Honeybees: Co- Infection by Aphid Lethal Paralysis, Israel Acute Paralysis and Lake Sinai Viruses. *Plos One* **8**, doi:10.1371/journal.pone.0057459 (2013).

47 Nielsen, S. L., Nicolaisen, M. & Kryger, P. Incidence of acute bee paralysis virus, black queen cell virus, chronic bee paralysis virus, deformed wing virus, Kashmir bee virus and sacbrood virus in honey bees (Apis mellifera) in Denmark. *Apidologie* **39**, 310-314, doi:10.1051/apido:2008007 (2008).
